# Supplementary material for: Skyrmions in Scalar Fields of Non‐Hermitian Optical Microcavities: Spontaneous Formation, Nonlinear Control, and Optical Forces
Source: Nanophotonics. 2026 Oct 9;15(19):e70303. doi: 10.1002/nap2.70303 (PMC13653164; doi:10.1002/nap2.70303)
Supplement: Supplementary file 1 [file NAP2-15-e70303-s001.pdf]

Supporting Information:
Skyrmions in scalar fields of non-Hermitian optical microcavities: spontaneous formation, nonlinear control, and optical forces

Jan Wingenbach,[1,2] Roman Lebs,[1] Xuekai Ma,[1] Ewan M. Wright,[3,4] Nai H. Kwong,[3] R. Binder,[3,4] Harald Giessen,[3,5] and Stefan Schumacher[1,6,3]

[1] *Department of Physics and Center for Optoelectronics and Photonics Paderborn (CeOPP), Paderborn University, 33098 Paderborn, Germany*
[2] *Institute for Photonic Quantum Systems (PhoQS), Paderborn University, 33098 Paderborn, Germany*
[3] *Wyant College of Optical Sciences, University of Arizona, Tucson, AZ 85721*
[4] *Department of Physics, University of Arizona, Tucson, AZ 85721*
[5] *Physikalisches Institut, Research Center SCoPE, and Integrated Quantum Science and Technology Center (IQST), University of Stuttgart, 70569 Stuttgart, Germany*
[6] *Institute for Photonic Quantum Systems (PhoQS), Paderborn University, 33098 Paderborn, Germany*

(Dated: September 2, 2026)

S1. LIGHT FORCES AND DISPLACEMENT FIELD RELATIONS

The displacement field of the stationary-mode polariton field ψ_s is defined as

$$R = \begin{pmatrix} \partial_x \psi_s \\ \partial_y \psi_s \\ \psi_s \end{pmatrix} \tag{S1}$$

and the polariton field is defined by

$$\psi_s(x,y) = A(x,y)\mathrm{e}^{i\phi(x,y)}\,, \tag{S2}$$

with an amplitude and phase distribution $A(x,y)$ and $\phi(x,y)$, as well as the polariton density $I(x,y) = |\psi_s(x,y)|^2 = A^2(x,y)$. Insertion into the displacement field provides

$$R = e^{i\phi} \begin{pmatrix} \partial_x A + iA\partial_x \phi \\ \partial_y A + iA\partial_y \phi \\ A \end{pmatrix} \tag{S3}$$

and respectively

$$\psi_s^* R = A \begin{pmatrix} \partial_x A + iA\partial_x \phi \\ \partial_y A + iA\partial_y \phi \\ A \end{pmatrix}\,. \tag{S4}$$

The in-plane displacement field R_2, i.e. the (x,y) components, then reads

$$\psi_s^* R_2 = A \begin{pmatrix} \partial_x A + iA\partial_x \phi \\ \partial_y A + iA\partial_y \phi \end{pmatrix}\,. \tag{S5}$$

Hence, we can write down the relation between the displacement field and the light forces:
The phase-gradient force is proportional to

$$F_\phi \propto I(x,y)\,\nabla\phi(x,y) = \Im\left\{\psi_s^*(x,y)\,R_2(x,y)\right\} \tag{S6}$$

and the intensity-gradient force is proportional to

$$F_I \propto \nabla I(x,y) = 2\Re\left\{\psi_s^*(x,y)\,R_2(x,y)\right\}\,. \tag{S7}$$

S2. RESONANT PUMP PROFILE DEFINITION

In this section, we provide the mathematical description of the resonant pump profile used to investigate both three-wave interference and moiré lattices. The resonant pump profile is described by

$$E(\mathbf{r},t) = E_0\, e^{i\omega f(t)} \sum_{j=1}^{N} \exp\left[-\frac{|\mathbf{r}-\mathbf{r}_j|^2}{2\sigma^2} + i\,\mathbf{k}_j\cdot\mathbf{r}\right], \tag{S8}$$

where

$$\mathbf{r} = (x,y), \qquad \mathbf{r}_j = (x_0(j), y_0(j)), \qquad \mathbf{k}_j = (k_x(j), k_y(j)).$$

E_0, σ, and $\omega = \frac{\hbar|\mathbf{k}|^2}{2m}$ are the amplitude, width, and frequency of each resonant pump spot. $f(t)$ holds the temporal envelope of the pump. $\mathbf{r}_j$ and $\mathbf{k}_j$ hold the positions of each pump spot in direct and reciprocal space.

S3. NON-RESONANT PUMP PROFILE DEFINITION

In this section we provide the mathematical description of the nonresonant pump profile used to investigate spontaneous Skyrmion formation in both three-wave interference and moiré lattices in polariton systems. The nonresonant pump profile is described by

$$P(x,y) = \sum_{m=0}^{M}\sum_{n=0}^{N} 0.5[1+\cos(k(x\cos(\alpha_{n,m})) + y\sin(\alpha_{n,m}))], \tag{S9}$$

with $\alpha_{n,m} = \theta_n + m\phi$ and

$$\theta_n = \frac{2\pi n}{3} \qquad M = 0, \qquad N = 5.$$

for the three-wave interference setting and

$$\theta_n = \frac{n\pi}{3}, \qquad M = 2, \qquad N = 5, \qquad \phi = 9.4^\circ.$$

for the moiré lattice setting.

S4. DETAILS ABOUT SKYRMION PATTERNS IN THREE-WAVE INTERFERENCE AND MOIRÉ LATTICES

Here, we show additional details about the arising Skyrmion patterns in the two excitation scenarios discussed in the main text. Around the system center, both resonant and nonresonant excitations lead to quantitatively identical results. Note that, as stated in the main part, nonresonant excitation allows for a more homogeneous density distribution, while the resonantly-driven interference patterns fade faster toward the outside. In Fig. S1 and S2, the results for three-wave interference and moiré lattice formation under resonant excitation are shown. In Fig. S3 and S4, the results for three-wave interference and moiré lattice formation under nonresonant excitation are shown. The panels show the wavefunction information, the in-plane displacement field, the full displacement field, and (in case of the three-wave interference) the spin density illustrating potential meron structures. These results are in very good agreement with the features measured in water and plasmonic systems as discussed in the main part of our work.

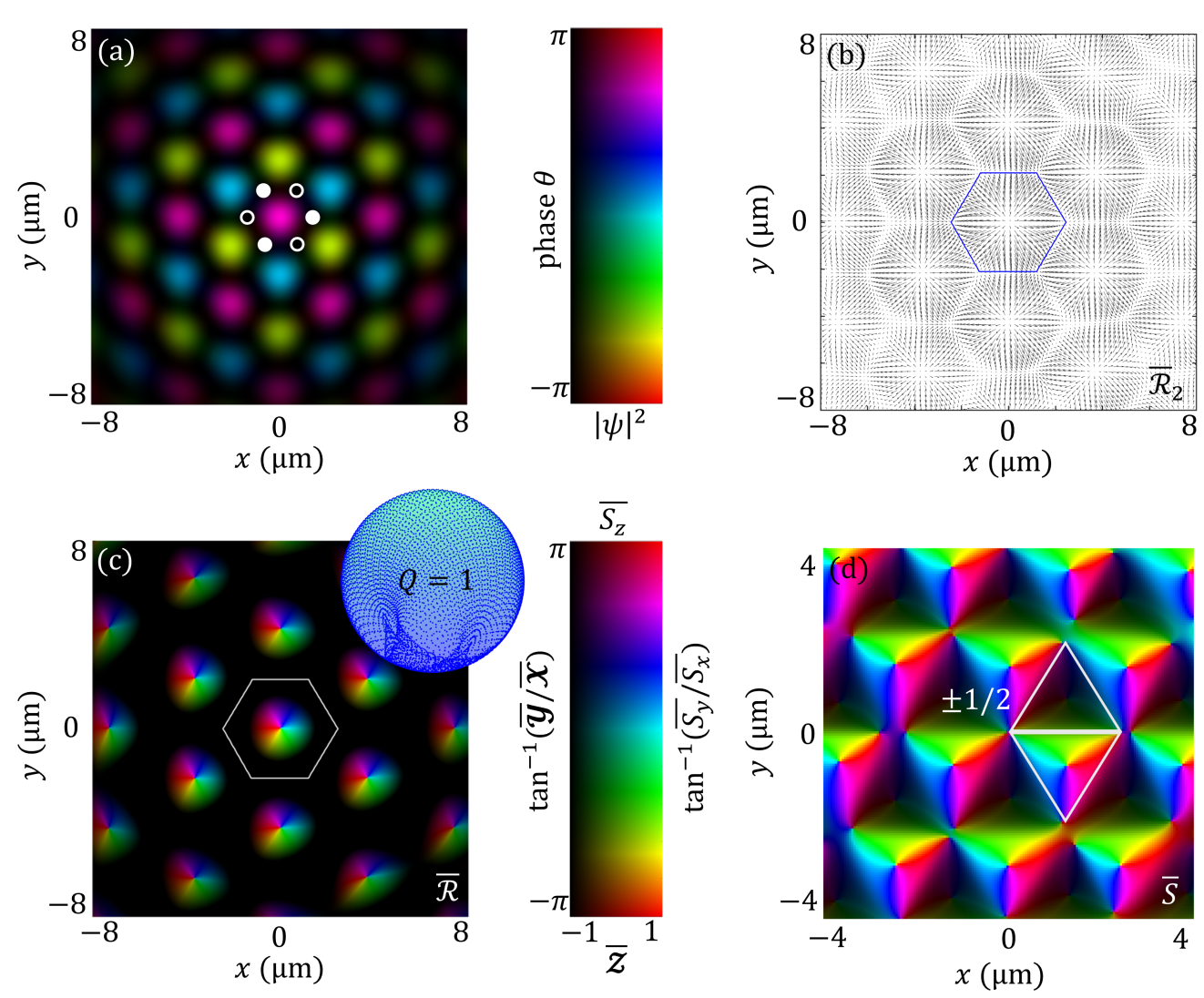

FIG. S1: **Skyrmion pattern under three-wave interference (resonant).** (a) Density (brightness) and phase (color) of the interference pattern. Vortex and anti-vortex pairs are highlighted by circles and dots. (b) In-plane displacement field, with Skyrmion cell marked in blue. (c) Full displacement field, with Skyrmion cell marked in white and unit-sphere mapping shown in the inset together with the Skyrmion number Q. (d) Spin density illustrating meron structure.

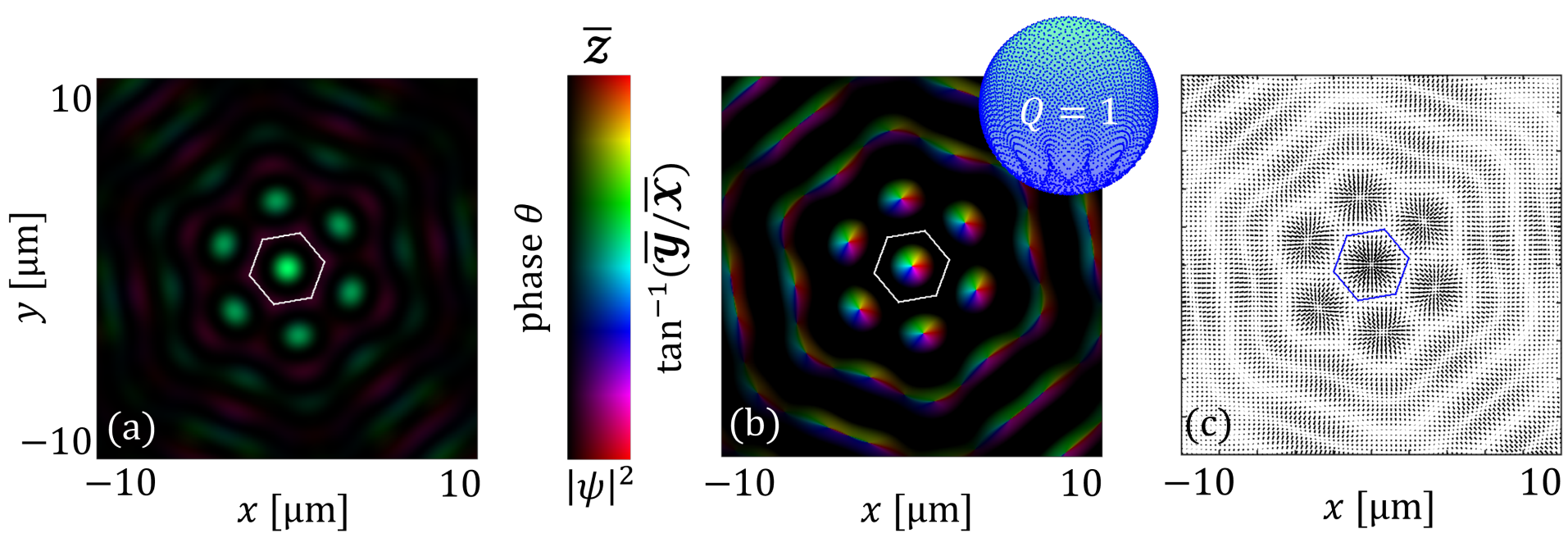

FIG. S2: **Skyrmion bag in moiré lattice (resonant).** (a) Density (brightness) and phase (color) of the interference pattern. (b) Full displacement field, with Skyrmion cell marked in white and unit-sphere mapping shown in the inset together with the Skyrmion number Q. (c) In-plane displacement field, with Skyrmion cell marked in blue.

S5. OPTICAL FORCES IN SKYRMION MOIRÉ LATTICE

In this section, the results for the optical forces for non-isolated Skyrmions are introduced. In Fig. S5 the optical forces on the Skyrmion moiré lattice are displayed. Notably, the density-gradient force F_I forms traps in the Skyrmion center, while the phase-gradient force F_ϕ permits trapping between Skyrmions.

S6. SIMPLIFIED THEORETICAL MODEL FOR SINGLE SKYRMION CASE

Here we present a simplified theoretical description of an isolated Skyrmion under nonresonant pumping, with the aim of enlightening the fundamental physical mechanisms underlying the solution. The starting equations are those in Eq. (1) of the main paper. For an incoherent pump P, we may set $E = 0$. Furthermore, we focus on threshold conditions, where $|\psi|^2 \to 0$ can be assumed while retaining a finite field amplitude ψ. For a cylindrically symmetric pump $P(r)$ we further seek an eigenstate for which

$$\psi(r,t) = e^{-i\mu t/\hbar} U(r),$$

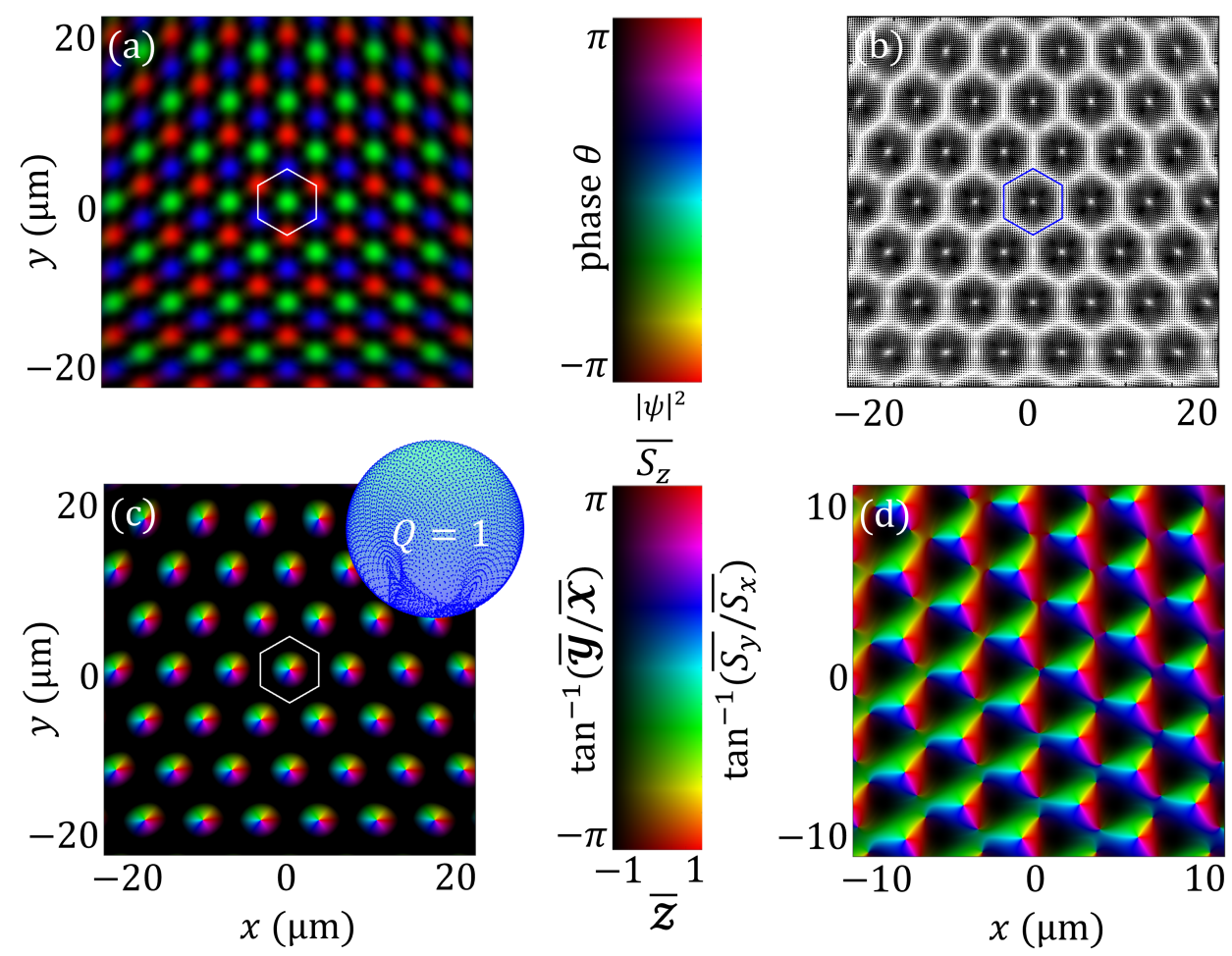

FIG. S3: **Skyrmion pattern under three-wave interference (nonresonant).** (a) Density (brightness) and phase (color) of the interference pattern. (b) In-plane displacement field, with Skyrmion cell marked in blue. (c) Full displacement field, with Skyrmion cell marked in white and unit-sphere mapping shown in the inset together with the Skyrmion number Q. (d) Spin density illustrating meron structure.

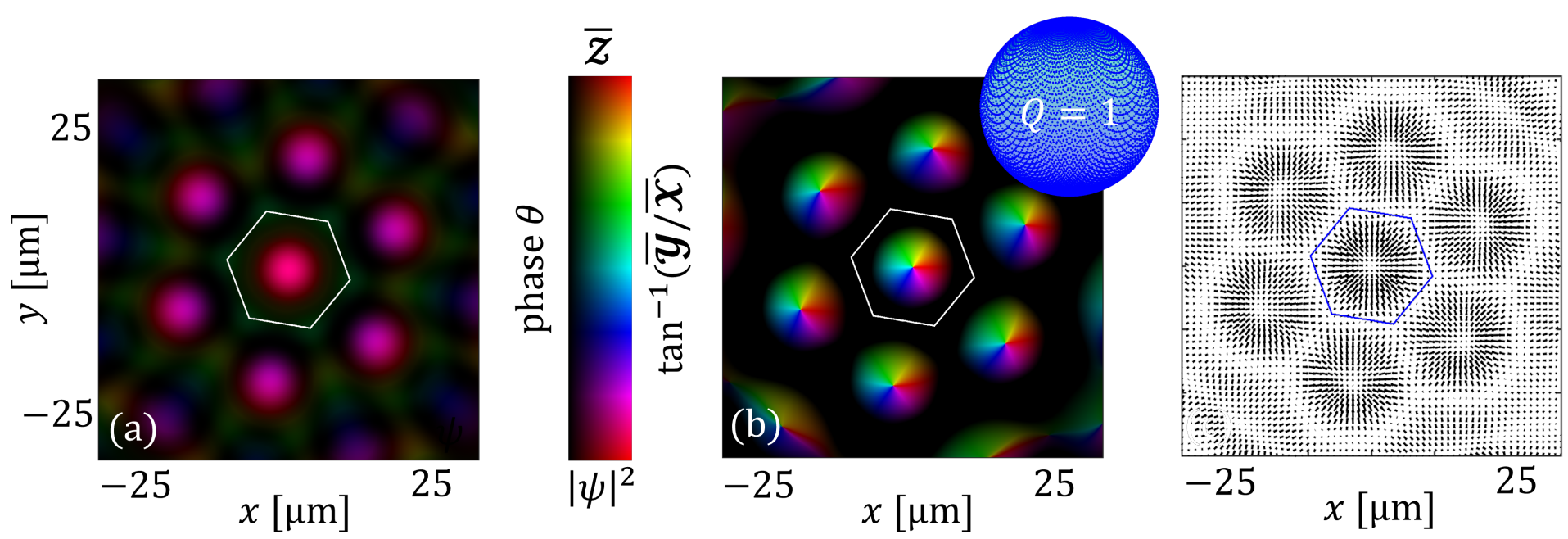

FIG. S4: **Skyrmion bag in moiré lattice (nonresonant).** (a) Density (brightness) and phase (color) of the interference pattern. (b) Full displacement field, with Skyrmion cell marked in white and unit-sphere mapping shown in the inset together with the Skyrmion number Q. (c) In-plane displacement field, with Skyrmion cell marked in blue.

with chemical potential μ. Under steady-state conditions, and neglecting nonlinear effects, the reservoir density is obtained as

$$n(r) = \frac{P(r)}{\gamma_r}.$$

The equation for the mode $U(r)$ then becomes

$$\mu U = \left(-\frac{\hbar^2}{2m}\nabla^2 + \frac{g_r}{\gamma_r}P(r) + \frac{i\hbar}{2}\left[\frac{R_c}{\gamma_r}P(r) - \gamma_c\right]\right)U. \tag{S10}$$

This non-Hermitian Schrödinger equation (NHSE) can be used to determine the threshold mode $U(r)$. The underlying motivation is that the threshold solution may already reveal key features of the system that persist above threshold, where nonlinear interactions become significant. Skyrmionic structures are known to exist even in passive externally driven cavities. The objective here is therefore to examine the corresponding incoherently pumped system at threshold, before nonlinear effects dominate the dynamics. In this capacity, we note that the chemical potential μ introduced above is complex to allow for loss and gain in the system. At threshold, the imaginary part of the complex chemical potential, $\mu = \mu' + i\mu''$, must vanish, ensuring an exact balance between gain and loss. Otherwise, the mode would either grow exponentially or decay in time.

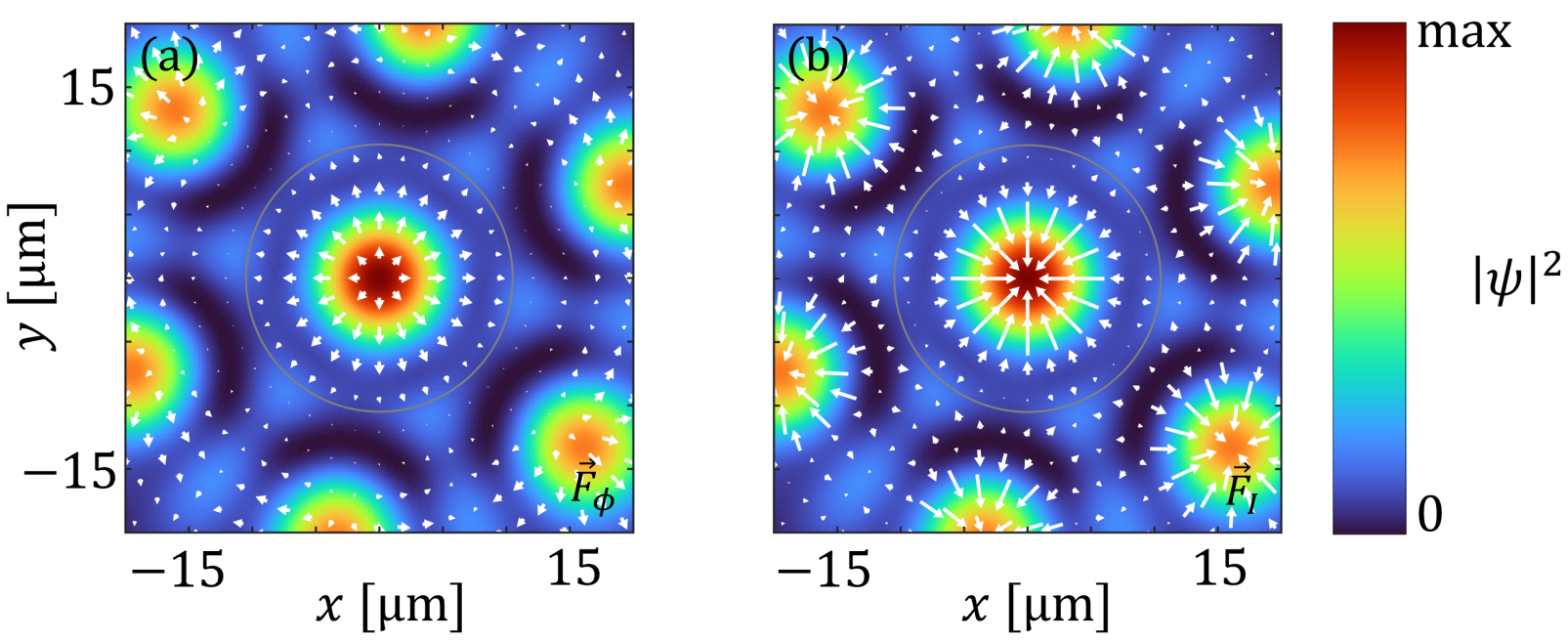

FIG. S5: **Optical forces in Skyrmion moiré lattice.** (a) Phase-gradient force and (b) Density-gradient force as vector fields in front of the density distribution (color) forming the Skyrmion moiré lattice. For visualization, the inner bag is displayed.

To derive an approximate analytical solution of the NHSE, we consider a Gaussian pump profile

$$P(r) = P_0 e^{-r^2/r_p^2} \approx P_0 \left(1 - \frac{r^2}{r_p^2} + \dots\right),$$

where P_0 denotes the peak pump intensity and r_p is the characteristic pump radius. A Taylor expansion around $r = 0$ is employed to describe the mode behavior near the center of the pump spot. Using the Taylor expansion eq. (S10) can be approximated as

$$\mu U \approx \left(-\frac{\hbar^2}{2m}\nabla^2 + \frac{g_r P_0}{\gamma_r}\left(1 - \frac{r^2}{r_p^2}\right) + \frac{i\hbar}{2}\left[\frac{R_c P_0}{\gamma_r}\left(1 - \frac{r^2}{r_p^2}\right) - \gamma_c\right]\right) U. \tag{S11}$$

A key observation is that the resulting equation can be mapped onto a quantum harmonic oscillator with a complex frequency, defined by

$$\frac{1}{2} m\omega_c^2 = -\frac{g_r P_0}{\gamma_r r_p^2} - \frac{i\hbar}{2}\frac{R_c P_0}{\gamma_r r_p^2}. \tag{S12}$$

Then, along with the definition

$$\mu^{(0)} = \frac{g_r P_0}{\gamma_r} + \frac{i\hbar}{2}\left[\frac{R_c P_0}{\gamma_r} - \gamma_c\right], \tag{S13}$$

the NHSE can be written as

$$\mu U = \mu^{(0)} U \underbrace{- \frac{\hbar^2}{2m}\left(\frac{d^2}{dr^2} + \frac{1}{r}\frac{d}{dr}\right) U + \frac{1}{2} m\omega_c^2 r^2 U}. \tag{S14}$$

The underbraced term in this NHSE is the complex quantum harmonic oscillator with an unnormalized Gaussian mode solution

$$U(r) = e^{-r^2/2r_c^2}, \quad r_c = \sqrt{\frac{\hbar}{m\omega_c}}, \tag{S15}$$

and complex eigenvalue $\hbar\omega_c$. In this context,

$$\frac{1}{r_c^2} = \frac{m\omega_c}{\hbar} = \frac{m\omega_c'}{\hbar} + i\frac{m\omega_c''}{\hbar},$$

must be chosen with the root such that $\omega_c' > 0$ so that the Gaussian mode

$$\begin{aligned} U(r) &= e^{-r^2/2r_c^2} \\ &= e^{-\frac{m\omega_c'}{2\hbar} r^2 - i\frac{m\omega_c''}{2\hbar} r^2}, \end{aligned} \tag{S16}$$

corresponds to a spatially confined mode. With this in mind, the complex chemical potential for the model is given by

$$\mu = \mu^{(0)} + \hbar\omega_c,$$

and the threshold condition $\mu'' = 0$ can be satisfied for a chosen set of parameters by suitable choice of γ_c.

The central feature of the problem is its non-Hermitian character and the resulting modification of the mode structure. For the usual real quantum harmonic oscillator, the modes can be chosen to have uniform phases, but here this is not possible even for the Gaussian mode, this is akin to the modes of so called gain guided semiconductor lasers where the oscillating mode has a phase curvature. The phase curvature associated with $\omega_c'' \neq 0$ plays a crucial role in enabling the Skyrmionic structure discussed below.

Next, we analyze the components of the real-valued displacement field and relate them to the behavior shown in Fig. 2 of the main manuscript for the Gaussian solution derived above. First, the density profile in Fig. 2(a) will have the radial variation $\propto e^{-\frac{m\omega_c'}{\hbar}r^2}$. The components of the real-valued displacement field (for $t = 0$) are:

- the x-component

$$\begin{aligned} \mathcal{X} &\propto \Re\left(\frac{\partial U}{\partial x}\right) \propto x e^{-\frac{m\omega_c'}{2\hbar}r^2}\left[\cos\left(\frac{m\omega_c''}{2\hbar}r^2\right) + \frac{\omega_c''}{\omega_c'}\sin\left(\frac{m\omega_c''}{2\hbar}r^2\right)\right] \\ &\propto x e^{-\frac{m\omega_c'}{2\hbar}r^2}\cos\left(\frac{m\omega_c''}{2\hbar}r^2 - \alpha\right), \end{aligned} \tag{S17}$$

  where $\alpha = \tan^{-1}\frac{\omega_c''}{\omega_c'}$,

- the y-component

$$\begin{aligned} \mathcal{Y} &\propto \Re\left(\frac{\partial U}{\partial y}\right) \propto y e^{-\frac{m\omega_c'}{2\hbar}r^2}\left[\cos\left(\frac{m\omega_c''}{2\hbar}r^2\right) + \frac{\omega_c''}{\omega_c'}\sin\left(\frac{m\omega_c''}{2\hbar}r^2\right)\right] \\ &\propto y e^{-\frac{m\omega_c'}{2\hbar}r^2}\cos\left(\frac{m\omega_c''}{2\hbar}r^2 - \alpha\right), \end{aligned} \tag{S18}$$

- and the z-component

$$\mathcal{Z} \propto \Re(U) \propto e^{-\frac{m\omega_c'}{2\hbar}r^2}\cos\left(\frac{m\omega_c''}{2\hbar}r^2\right). \tag{S19}$$

In each component, the trigonometric terms account for the radial sign reversals observed in the field distribution, as illustrated for $\mathcal{Z}$ in Fig. 2(c) of the main text. Similarly, the prefactors appearing in $\mathcal{X}$ and $\mathcal{Y}$ have the form of first-order Hermite-Gaussian modes along the x and y directions. This explains why $\mathcal{R}_2$ in Fig. 2(d) vanishes at the origin, while the cosine modulation accounts for the additional nodal ring structure. The Gaussian approximation should not be interpreted quantitatively for radii significantly larger than the pump radius r_p. This limitation arises from the parabolic approximation of the gain profile, which introduces nonphysical absorption at sufficiently large distances from the pump center.

The purpose of the simplified model is to demonstrate that the Skyrmionic character of the solutions is intimately connected to the distinctive modal properties of the NHSE. In particular, the analysis links the emergence of Skyrmionic features to the phase curvature of the non-Hermitian eigenmodes, a quantity that is, in principle, experimentally accessible. The model is not intended to provide quantitatively precise predictions; rather, its value lies in the physical insight it offers and in its potential to reveal how the modal properties scale with the relevant system parameters. Although the approach could be extended through a variational Gaussian treatment, such a refinement is unlikely to yield substantially deeper physical insight.

S7. SKYRMION NUMBER AS A FUNCTION OF SKYRMION CELL RADIUS

Here we discuss the scaling of the Skyrmion number with the radius of the circular integration domain in case of the single spontaneous Skyrmion formed in Fig. 2 of the main manuscript. To visualize the dependence of the

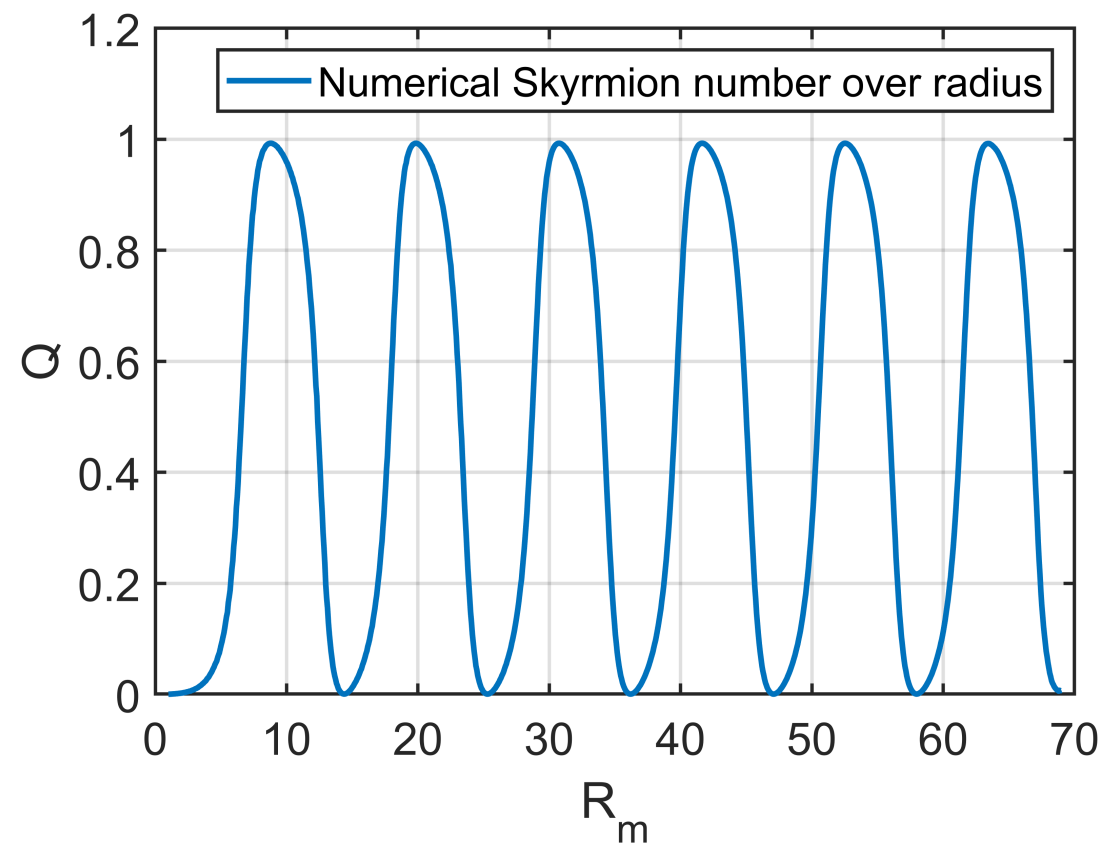

FIG. S6: Calculated Skyrmion number Q as a function of the Skyrmion cell radius R_m. Due to the normalization of the displacement field, the resulting topological charge oscillates between $Q = 0$ and $Q = 1$ depending on the selected integration radius.

Skyrmion number on the chosen integration domain size, Figure S6 shows the cell radius as a function of the resulting Skyrmion number. The Skyrmion number was calculated using the normalized displacement field. The computed value oscillates between $Q = 0$ and $Q = 1$ depending on the selected radius.

Importantly, the radius used for the Skyrmion number calculation is not chosen arbitrarily, nor is it adjusted iteratively until $Q = 1$ is obtained. Instead, the radius is determined from the first minimum of the displacement field component $\mathcal{Z}$. In practice, the extrema of the displacement field are used to define the boundaries of the Skyrmion cell, ensuring that the smallest physically meaningful cell is selected for the calculation.
